# The Characterization of Polymers That Mimic the Aortic Wall’s Mechanical Properties and Their Suitability for Use in the 3D Printing of Aortic Phantoms

**DOI:** 10.3390/polym17121700

**Published:** 2025-06-19

**Authors:** Moritz Wegner, Benan Sahin Karagoez, David Wippel, Florian K. Enzmann, Anja Niehoff, Oroa Salem, Bernhard Dorweiler

**Affiliations:** 1 Department of Vascular and Endovascular Surgery, Faculty of Medicine, University Hospital Cologne, University of Cologne, Kerpener Strasse 62, 50937 Cologne, Germany; 2 Department of Vascular Surgery, Medical University Innsbruck, Anichstrasse 35, 6020 Innsbruck, Austria; 3 Institute of Biomechanics and Orthopaedics, German Sport University Cologne, 50933 Cologne, Germany; 4 Cologne Center for Musculoskeletal Biomechanics, Faculty of Medicine, University Hospital Cologne, University of Cologne, Kerpener Strasse 62, 50937 Cologne, Germany

**Keywords:** printing, three-dimensional, material testing, simulation training, polymers, aortic diseases

## Abstract

(1) While three-dimensional (3D) printing technology is increasingly being used for the fabrication of high-fidelity, patient-specific aortic models, data on the mechanical properties of polymers are sparse. Therefore, the aim of this study was to identify suitable polymers for this purpose. (2) Methods: Eight flexible polymers, with Shore A hardnesses (ShA) of 27–85, were tested to determine their suitability for PolyJet printing technology. They were tested against porcine aortic and bovine pericardial tissue for suture retention strength, uniaxial stress testing according to ISO 37, and burst pressure in a standardized test setting. (3) Results: The polymers with a ShA of 30–50 showed statistically non-inferior suture retention strength, tensile strength, and burst pressure resistance when compared to pericardial and aortic tissue, respectively. (4) Conclusions: This was the first report to analyze the mechanical properties of eight different flexible PolyJet polymers. We found that the polymers with a Shore A hardness of 30–50 most closely mimicked the mechanical properties of aortic tissue. Therefore, they can be recommended for the additive manufacturing (3D printing) of aortic phantoms for simulation and training purposes.

## 1. Introduction

Anatomical modeling and medical simulations have become integral components of modern surgical planning, procedural training and patient education, and three-dimensional (3D) printing technology has been instrumental in enabling the fabrication of patient-specific anatomical models. High-fidelity models offer realistic and reproducible environments for simulating complex interventions, optimizing surgical strategies, enhancing surgical training, and assessing novel technologies prior to clinical application [1,2,3,4,5,6,7].

In vascular surgery, there is a pronounced demand for models of vessel pathology that are both realistic and reproducible, because the success of a procedure is contingent upon precise vessel navigation and mechanical fidelity [8,9,10]. PolyJet printing has emerged as a promising technology for producing tissue models for vascular applications due to its high resolution, multi-material capability, and its tunable mechanical properties [11,12,13]. PolyJet photopolymers, such as Agilus^®^- and Tango^®^-based materials (Stratasys, Rehovot, Israel), can facilitate the precise modulation of mechanical properties, thereby enabling the replication of vascular tissues with a high degree of fidelity [14,15,16]. However, while previous studies have characterized the general mechanical properties of PolyJet photopolymers, their capacity to realistically mimic human vascular biomechanics remains insufficiently explored and controversial [17,18,19,20].

The objective of this study was to provide a systematic biomechanical evaluation of PolyJet-printed photopolymers, including tests of their tensile strength, burst pressure resistance, and suture retention strength. Additionally, we compared the properties of these photopolymers to those of commonly used biological reference materials, including porcine aorta and bovine pericardium, to identify the optimal materials for creating high-fidelity aortic phantoms.

## 2. Materials and Methods

### 2.1. Polymers

In this study, the properties of eight different polymers of a proprietary chemical composition (Stratasys, 1 Holtzman St. Science Park, P.O. Box 2496 Rehovot 7612401, Israel) and with Shore A hardnesses (ShA) ranging from 27 to 85 were tested to assess their suitability for object-printing technology (UV-cured): Two of the polymer solutions were derived from single materials: TangoPlus^®^ FLX930 (ShA 27) and Agilus30^®^ clear (ShA 30), whereas the six remaining polymers (ShA 35–85) were composed during the printing process by mixing two polymer solutions (Agilus30^®^ clear and VeroClear^®^) in the following (proprietary) ratios: FLXA-MT-S35-DM (ShA 35), FLXA9940-DM (ShA 40), FLXA9950-DM (ShA 50), FLXA9960-DM (ShA 60), FLXA9970-DM (ShA 70), and FLXA9985-DM (ShA 85). After printing, the patches were removed from the printing tray, cleaned, and stored in de-humidified boxes until they were used.

For each polymer, patches (width 60 mm, length 130 mm, and thickness 2 mm) were manufactured using additive technology and a high-precision Polyjet printer (Objet 350 Connex 3, Stratasys, Rehovot, Israel), with a spatial printing resolution of 30 µm. From each patch, 10 test pieces were cut, as described below.

### 2.2. Control Tissues

The bovine pericardium patch (Supple Peri-Guard, Baxter/Synovis Life Technologies, Unterschleissheim, Germany) was used as a control material. From each patch (8 × 14 cm), 10 test pieces were cut, as described below. Supple Peri-Guard^®^ is prepared from bovine pericardium procured from cattle that originate in the United States and is cross-linked with glutaraldehyde. Supple Peri-Guard is chemically sterilized using ethanol and propylene oxide and is treated with 1 molar sodium hydroxide for 60–75 min at 20–25 °C.

Pieces of porcine aortic tissue (thoracic and abdominal) were collected at a local slaughterhouse from domestic pigs aged 6–12 months. The aortae were cut along the longitudinal axis, and the dumb-bell specimens were cut in parallel to the longitudinal direction of the aortic tube. The porcine aortic tissue was stored at 6–8 °C in a physiologic saline solution, and it was used in a timely manner.

### 2.3. Generation of Test Pieces

First, the suture retention strength of a single stitch was analyzed. For this purpose, a single polypropylene cross-stitch (Prolene^®^, 5-0 metric SH, Ethicon/Johnson & Johnson Medical GmbH, Norderstedt, Germany) was placed 2 mm from the margin of a rectangular test piece (2 × 1 cm) as described earlier [21].

**Figure 1:**
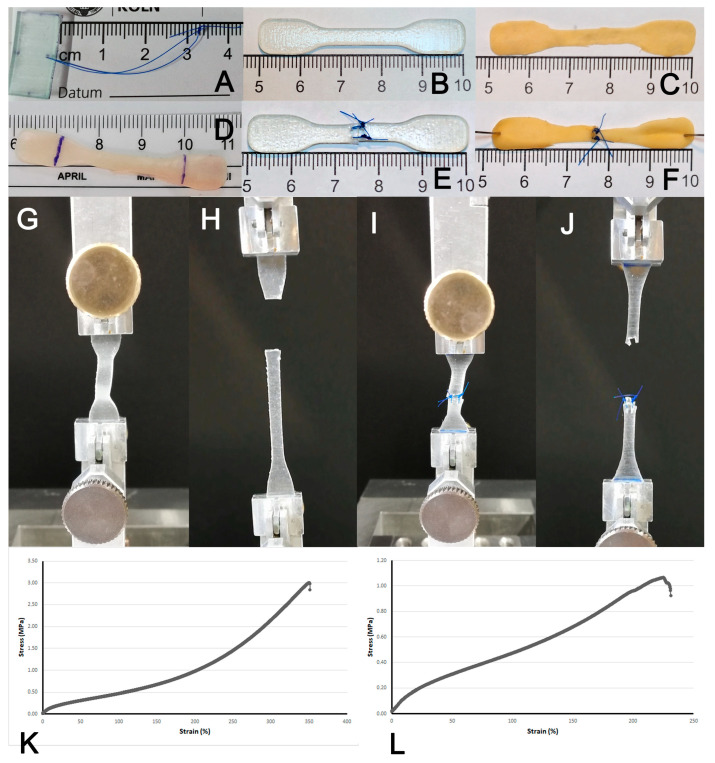
Test setting for uniaxial stress tests. Test pieces for assessment of suture retention strength (**A**) and stress–strain testing with intact ((**B**) polymer, (**C**) pericardium, (**D**) aortic tissue) and sutured ((**E**) polymer, (**F**) pericardium) patches (Type 3/ISO 37). Appearance of the testing machine (Zwick Roell Z2.5/TN1S) at start ((**G**) intact, (**I**) sutured) and end position ((**H**) intact, (**J**) sutured). Representative stress–strain curve for intact (**K**) and sutured (**L**) polymer.

Second, dumb-bell-shaped test pieces (dimension: length 50 mm) were cut according to Type 3/ISO 37 [22]. from the polymer patches, bovine pericardium and porcine aortic tissue, respectively, and used for the experiments immediately (Figure 1) [21].

For the simulation of sutured material, the dumb-bell test pieces were cut in the middle and then sutured back together using two interrupted atraumatic polypropylene sutures (Prolene^®^, 5-0 metric SH) placed 1 mm from the lateral margin of the center part and 2 mm from the cutting plane of the test piece (4 mm diameter), respectively.

Thickness of polymer pieces as well as aortic and bovine pericardial tissue was determined by a digital high-fidelity (precision 0.01 mm) caliper (DigiMax^®^, WIHA, Schonach, Germany) and revealed mean thicknesses of 2.0 ± 0.08 mm for polymer pieces, 0.5 ± 0.09 mm for pericardial patches, and 1.2 ± 0.11 mm for aortic pieces.

### 2.4. Suture Retention Strength, Uni-Axial Stretch and Burst Pressure Testing

Testing of suture retention strength and uni-axial stretch testing were performed with a Zwick Roell Z2.5/TN1S material testing machine (Zwick GmbH & Co. KG, Ulm, Germany) at standardized conditions (ISO 37). The test pieces were placed in the clamps of the testing machine as indicated by the manufacturer (Figure 1). Primary data collection encompassed elongation (in mm) and force (in N). For calculation of tensile strength, the maximum value of force was retrieved for each experiment.

**Figure 2:**
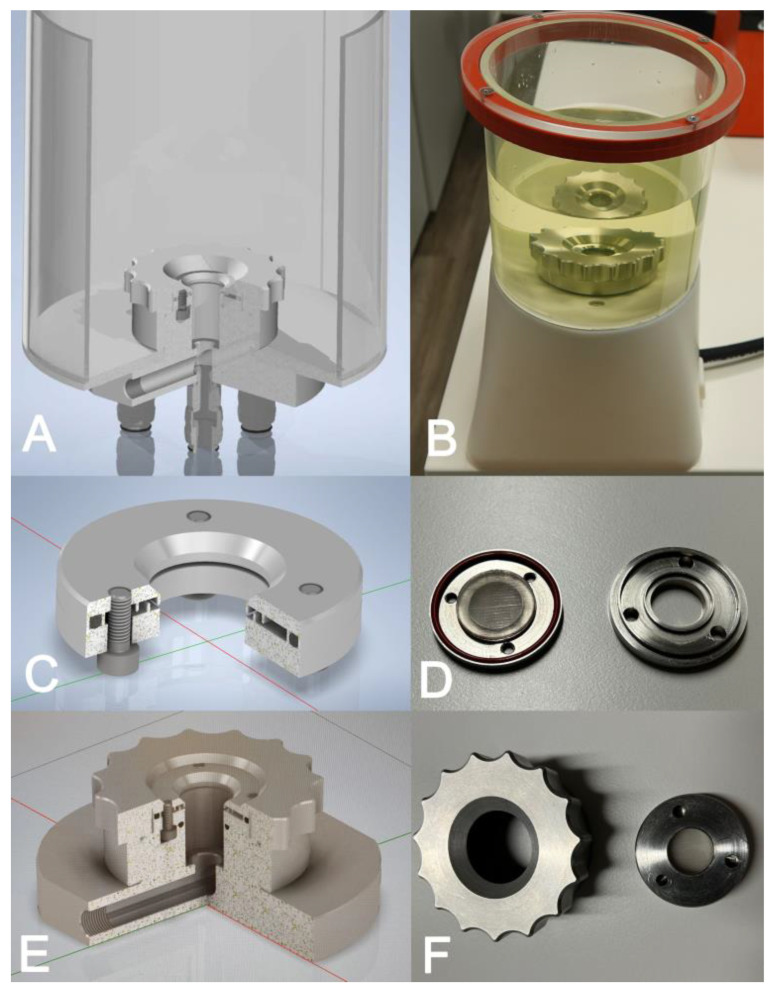
Test device for assessment of burst pressure resistance. A test device consisting of a steel pressure chamber that is attached to a polymethylmethacrylat housing (**A**) covered with a polymethylmethacrylat lid and mounted on a stand (**B**) was manufactured (core engineering facility, University of Cologne). The polymer/tissue patch was placed within the two-piece steel test chamber (**C**,**D**), which then was fixed to the base using a screw cap (**E**,**F**). Images (**A**,**C**,**E**) show the engineering sketch; images (**B**,**D**,**F**) show the final parts.

For burst pressure experiments, a pressure chamber was constructed (adopted from Chen et al. 2016 [23]) and manufactured (Scientific Workshop, Faculty of Medicine, University of Cologne) (Figure 2). It consists of a circular steel base with a connection to the pressure line and a top screw that both fixate a testing disc. The latter is composed of two circular parts for fixation of the test material (circular patch, 25 mm diameter) and is secured with three screws. The pressure housing itself was placed in a plastic container covered with a lid to retain the water and allow for safe testing conditions. Finally, a digital pump for water pressure testing (RP50 digital, Rothenberger GmbH, Kelkheim, Germany) was connected to the self-made burst pressure apparatus/housing. The pressure (kPa) and time were recorded and the maximum value at burst was noted as indicated.

For imaging purposes of the burst pressure experiments, the pressurized solution was supplemented with Uranin^®^ UV-fluorescent dye (Hansepro GmbH, Garrel, Germany) and the imaging was acquired under UV light in selected representative cases (cases were not included in the experimental dataset).

### 2.5. Statistical Analysis

All experiments were carried out in 10 repetitions. For variables with normal distribution, mean and standard deviation were calculated, while for other variables, median and range were shown. Statistical testing was carried out by using the Mann–Whitney U test (2 groups) or the ANOVA with Kruskal–Wallis test (>2 groups). In either case, a *p*-value < 0.05 was considered significant. Statistical analysis was performed with GraphPad Prism 3 (GraphPad Software Inc., San Diego, CA, USA).

## 3. Results

### 3.1. Assessment of Suture Retention Strength

**Figure 3:**
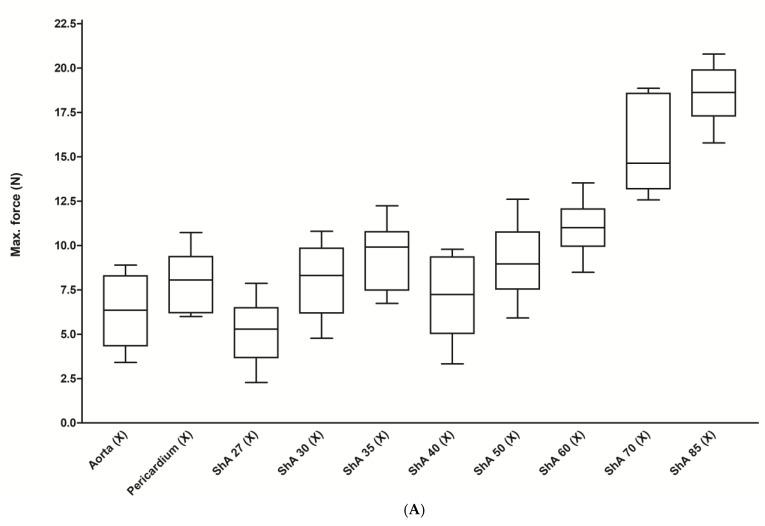

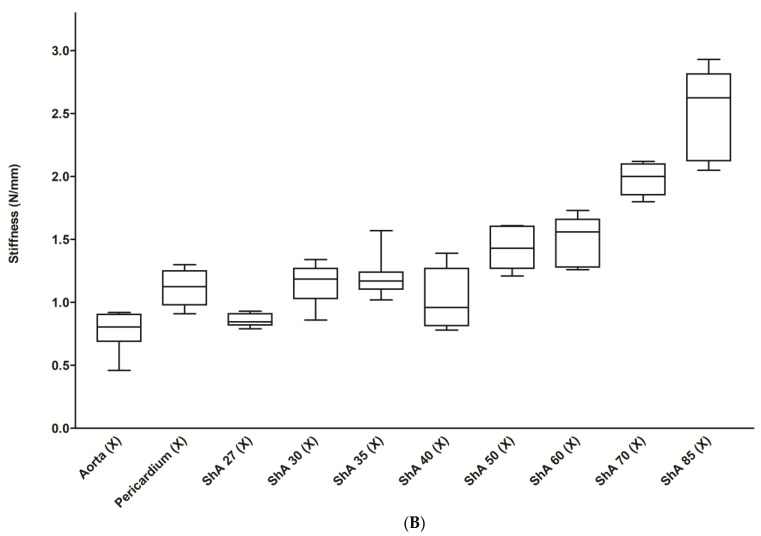
Results of test for suture retention strength. Maximum pullout force (**A**) and stiffness (**B**) were measured using a rectangular test piece with one polypropylene stitch (X).

For the initial screening tests, we used a simplified method of assessing the suture retention strength of a single monofilament (5-0) cross-stitch (X) (Figure 3A). Here, we were able to show that aortic and pericardial tissue had a statistically similar maximum force of 6.3 ± 2.0 N and 8.0 ± 1.6 N (*p* = 0.063), respectively. All polymers tested (ShA 27–85) proved a statistically similar or even higher pullout force ranging from 5.2 ± 1.7 to 18.6 ± 1.6 N (mean 10.5 ± 4.5 N). The same was true when stiffness was assessed, with a mean value of 0.8 ± 0.14 N/mm for aortic tissue and a range of 0.9 ± 0.05 to 2.5 ± 0.32 N/mm for all polymers (ShA 27–85) (Figure 3B).

However, due to the experimental setting, only the maximum force and stiffness could be evaluated in those experiments. Therefore, we performed additional mechanical testing experiments (stress–strain analysis) using a standardized setting according to international standard ISO 37. These experiments were conducted on a universal testing machine.

### 3.2. Stress–Strain Analysis

**Figure 4:**
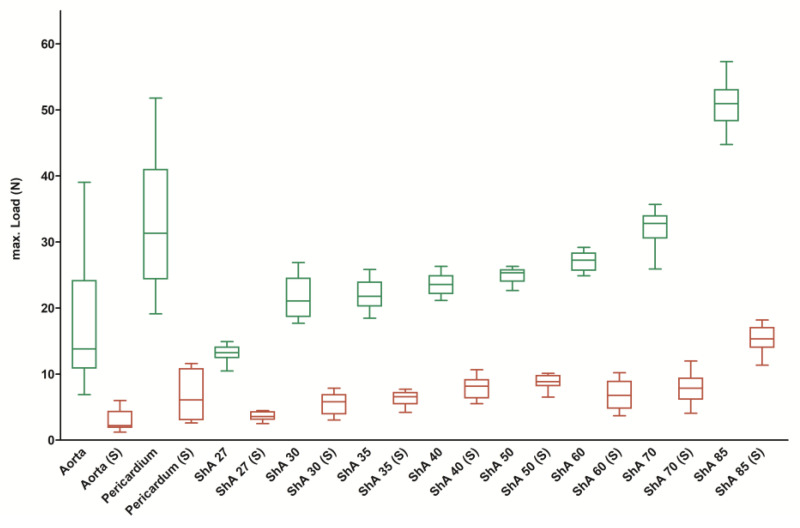
Assessment of maximum force. Load–deformation experiments were conducted using intact (green boxes) standardized bone-shaped (dumb-bell) testing pieces as well as sutured testing pieces (S, red boxes).

For the intact pieces, the maximum force of aortic tissue was 17.8 ± 11.1 N (Figure 4). The values of maximum force for the eight polymers tested ranged from 13.1 ± 1.3 N (ShA 27) to 50.9 ± 3.4 N (ShA 85) and were not statistically different from aortic tissue for ShA 27–60. ShA 70 and ShA 85, however, showed a significantly higher maximum force (*p* = 0.0117 and *p* = 0.0001, respectively). Pericardial tissue had a significantly higher maximum force (32.9 ± 10.0 N) when compared to aortic tissue (*p* = 0.0117).

**Figure 5:**
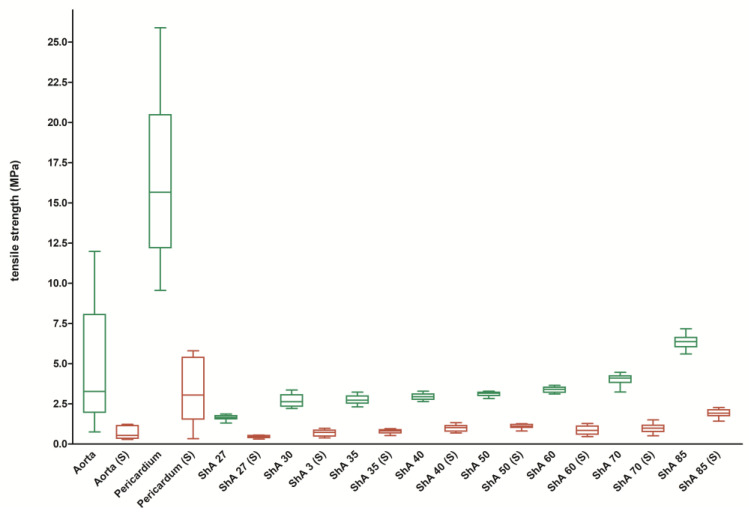
Assessment of tensile strength. Stress–strain experiments were conducted using intact (green boxes) standardized bone-shaped (dumb-bell) testing pieces as well as sutured testing pieces (S, red boxes).

When tensile strength was analyzed (Figure 5), aortic tissue showed a value of 4.6 ± 3.7 N/mm^2^, whereas the polymers ranged from 1.7 ± 0.16 MPa (ShA 27) to 6.4 ± 0.4 MPa (ShA 85). While polymers ShA 30–85 had similar values of tensile strength, ShA 27 showed significantly reduced tensile strength (*p* = 0.0098) when compared to aortic tissue.

**Figure 6:**
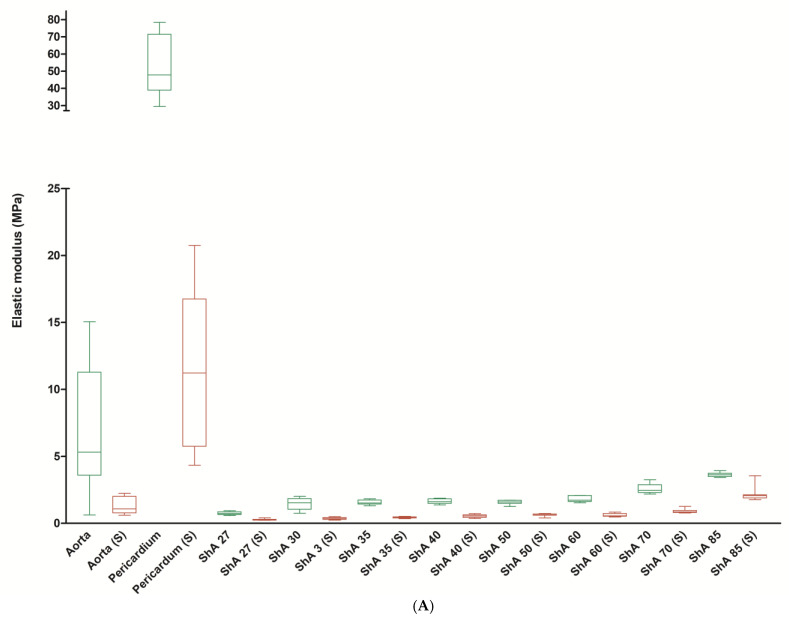

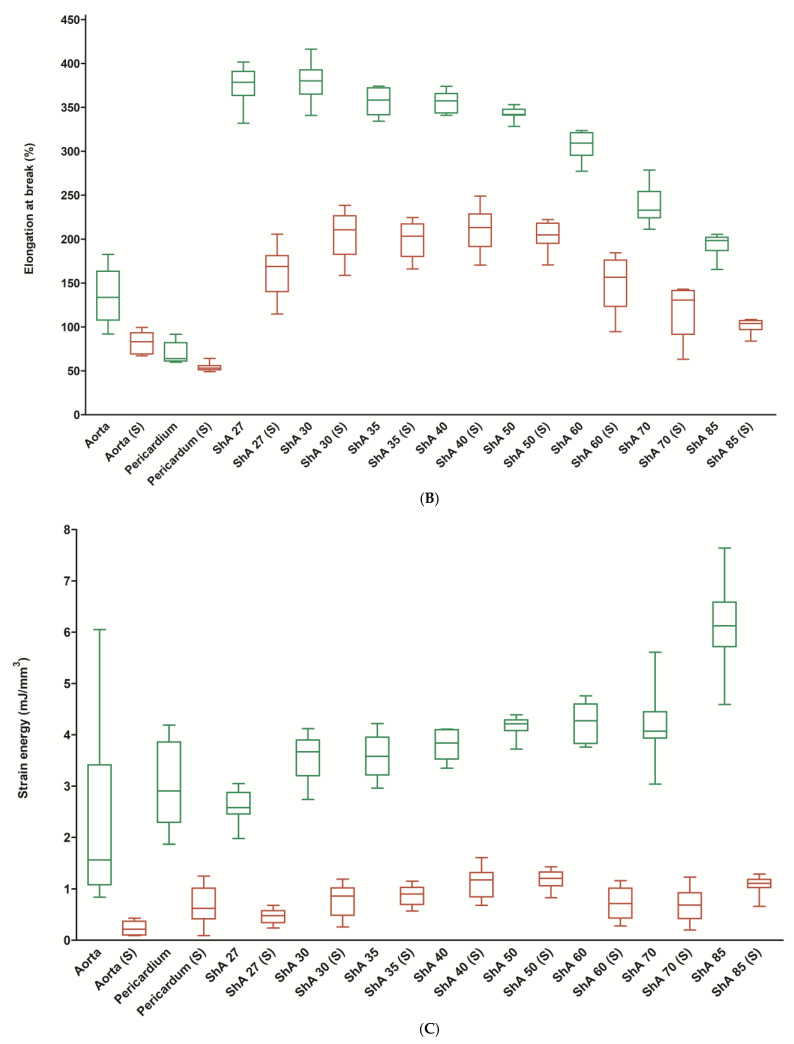
Assessment of elastic modulus, elongation at break, and strain energy. Elastic modulus (**A**), elongation at break (**B**) and strain energy (**C**) were analyzed using intact (green boxes) standardized bone-shaped (dumb-bell) testing pieces as well as sutured testing pieces (S, red boxes).

The elastic modulus describes the resistance of a material to being deformed elastically (i.e., non-permanently) when a stress is applied to it and is defined as the slope of its stress–strain curve in the elastic deformation region. Here, it was evident that aortic tissue and, even more pronounced, pericardial tissue had a much higher elastic modulus of 6.8 ± 4.5 MPa and 54.1 ± 17.9 MPa when compared to the polymers, which ranged from 0.8 ± 0.1 MPa (ShA 27, *p* = 0.0011) to 3.6 ± 0.2 MPa (ShA 85, *p* = 0.0355) (Figure 6A). Within the polymers, ShA 30–50 exhibited a similar elastic modulus (*p* = 0.6842). The results of elastic modulus are in turn corroborated by analysis of elongation at break. Here, aortic tissue could be extended to 134% ± 29.6% (compared to original test piece length) until break (Figure 6B). The polymers, however, were in a range of 376% ± 19.9% (ShA 27) to 194% ± 12.0% (ShA 85), thus all significantly higher than aortic tissue (*p* = 0.0001). Finally, we analyzed strain energy, which represents the area under the stress–strain curve and indicates the amount of energy that can be stored in a material upon deformation until the point of break (Figure 6C). Interestingly, aortic tissue (2.4 ± 2.0 mJ/mm^3^), pericardial tissue (2.9 ± 0.8 mJ/mm^3^), and polymer ShA 27 (2.6 ± 0.3 mJ/mm^3^) show similar values (*p* = 0.1011). All the remaining polymers (ShA 30–85) proved non-inferiority with significantly higher values for strain energy (range 3.6 ± 0.4 to 6.1 ± 0.8 mJ/mm^3^).

In order to assess the suitability of the different materials for suturing, which represents an integral feature for their use in simulation and training, we created a standardized model of suturing on the dumb-bell test pieces (annotated with (S)) and subjected them to the above-described analyses.

It became evident that cutting/suturing resulted in a marked decrease of mechanical behavior of the polymers and maximum force dropped to values between 3.6 ± 0.6 N (ShA 27 (S)) and 15.3 ± 1.9 N (ShA 85 (S), *p* = 0.0001) (Figure 4). However, the same effect was observed for aortic tissue (2.9 ± 1.7 N) and pericardial tissue (6.6 ± 3.4 N, *p* = 0.0001). But again, the maximum force for each of the sutured polymers was not inferior to sutured aortic tissue (*p* = 0.0789 for ShA 27 and *p* = 0.0041 for ShA 30 and above).

**Figure 7:**
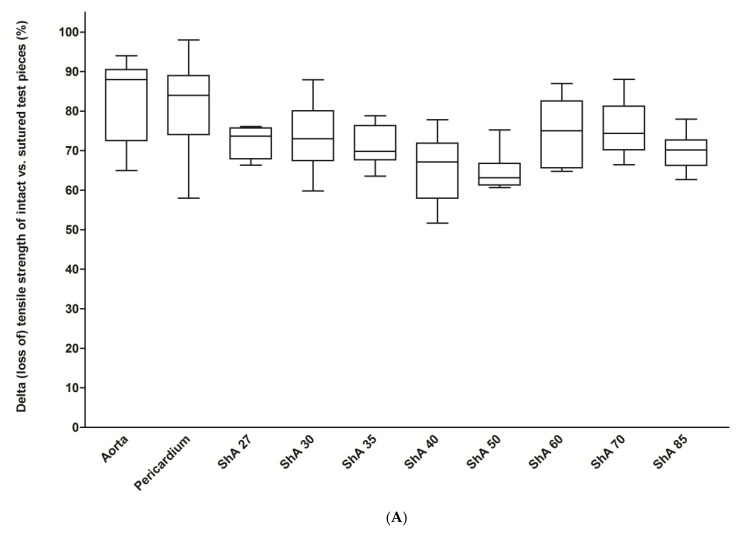

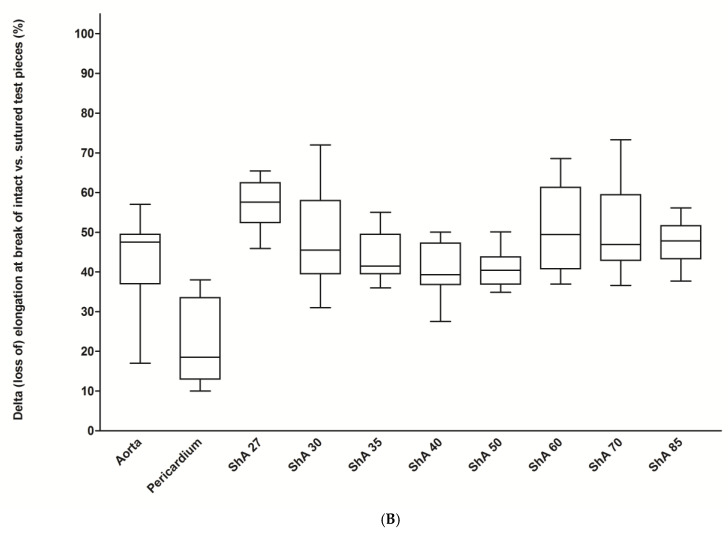
Impact of suturing on tensile strength and elongation at break. For all materials tested, a differential value (in %) was calculated that indicates loss of tensile strength (**A**) and decrease in elongation at break (**B**) for sutured vs. intact patches of the respective material.

Furthermore, suturing resulted in a mean reduction/loss of tensile strength of 71% for all polymers but 83% for aortic and 82% for pericardial tissue (Figure 7A). However, the reduction in the polymers’ tensile strength was significantly lower compared to aortic and pericardial tissue (0.0004 < *p* < 0.035, except for ShA 30 (*p* = 0.0535) and ShA 70 (*p* = 0.1330)). Similar to tensile strength, the elastic properties were altered by suturing. Here, suturing resulted in a mean reduction in elongation at break of 47% for all polymers (range: 41% to 57%) (Figure 7B). Aortic tissue showed a reduction of 43%, while pericardial tissue showed a reduction of only 22% compared to intact tissue (*p* = 0.0021). Of note is that, when compared to aortic tissue, the reduction in elongation at break for all polymers tested was statistically similar (except for ShA 27, *p* = 0.0044), and thus not inferior.

Finally, suturing of the test pieces also affected strain energy and elastic modulus of the materials. For both parameters, a significant decrease was noted for aortic and pericardial tissue as well as for the polymers. The elastic modulus was reduced to 1.3 ± 0.6 mJ/mm^3^ for aortic tissue and 11.5 ± 5.7 mJ/mm^3^ for pericardial tissue. All polymers showed a significant reduction, too (Figure 6A). However, the strain energy of the polymers (i.e., ShA 27) was still significantly higher compared to aortic tissue (0.5 ± 0.1 vs. 0.2 ± 0.1 mJ/mm^3^, *p* = 0.0015) (Figure 6C).

**Figure 8:**
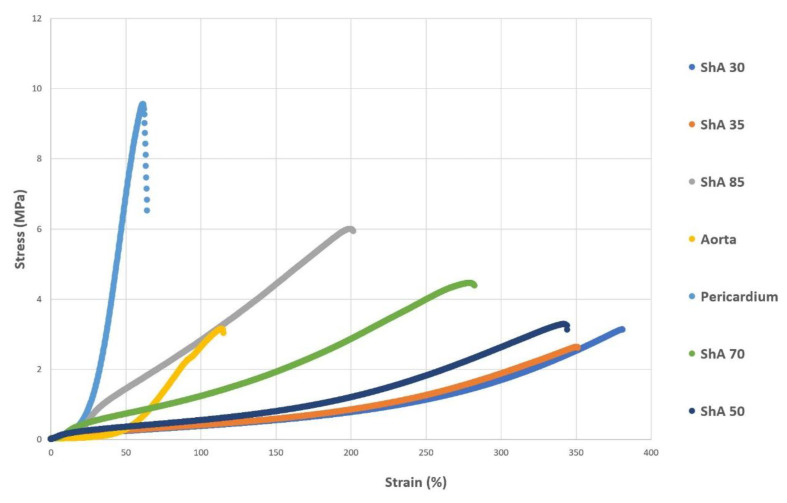
Cumulative curve plots for stress–strain analysis. Polymers (ShA 30–85) and control tissues (porcine aorta and bovine pericardium) were subjected to stress–strain analysis and results plotted in one diagram.

A comparative analysis of stress–strain diagrams for polymers and control tissue (Figure 8) confirmed that in a range of up to 50% strain (which resembles a meaningful working scenario for simulation and training purposes using the respective materials), polymers of low Shore hardness (ShA 30 to ShA 50) show a similar curve plot compared to aortic tissue. In contrast, pericardial tissue and polymers of higher Shore hardness (> ShA 60) show a markedly different curve plot.

### 3.3. Burst Pressure Testing

**Figure 9:**
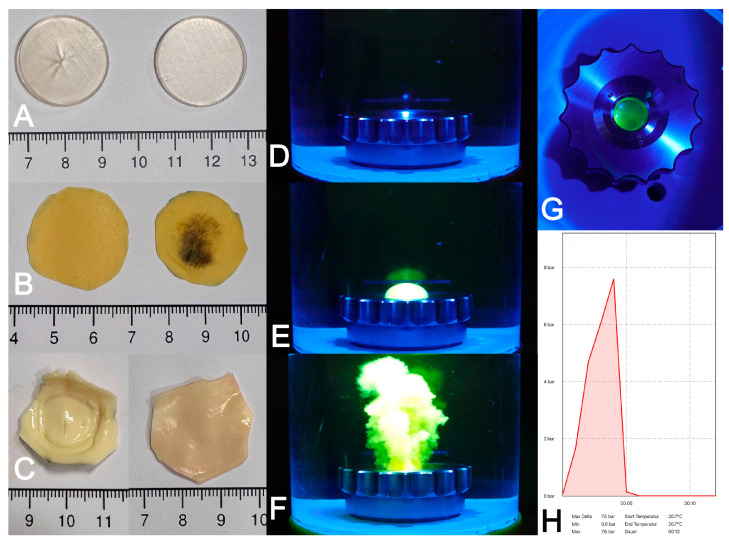
Results of burst pressure testing. Circular test patches before and after the burst pressure test ((**A**) polymer, (**B**) pericardium, (**C**) aortic tissue). A representative image sequence of a pressure test demonstrating baseline at start (**D**), bulging (**E**) and rupture (**F**) of the patch. View from above on the test chamber (**G**) and representative pressure curve of a burst pressure experiment (**H**).

For burst pressure testing, circular patches made from the different materials were subjected to increasing pressure in a self-made apparatus. Prior to and after the test, the specimens were checked for structural integrity, thereby ensuring that the perforation site was located in the center of the patch (Figure 9). During the tests, the respective pressure/time diagrams and the maximum value of pressure at burst were recorded and used for further analysis.

**Figure 10:**
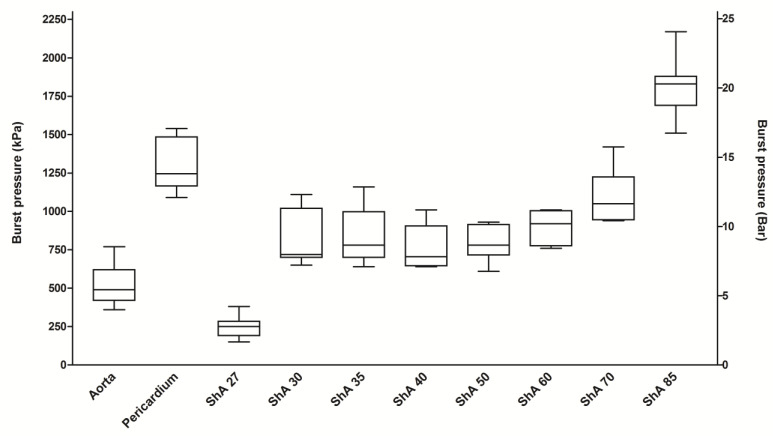
Assessment of burst pressure. Circular patches of the polymers (ShA 27–85) and control tissues (porcine aorta, bovine pericardium) were subjected to increasing pressure in a pressure chamber and the pressure at burst (peak of pressure curve) was recorded.

Aortic tissue withstood a mean pressure of 520 ± 135 kPa until rupture (Figure 10). In contrast, bovine pericardium showed a significantly higher burst pressure of 1303 ± 176 kPa (*p* = 0.0001). The polymers having Shore hardness of 30–85 also withstood a significantly higher burst pressure when compared to aortic tissue (*p* < 0.0176). However, the polymer with a Shore hardness of 27 (TangoPlus^®^) showed a significantly lower pressure resistance of only 247 kPa when compared to aortic tissue (*p* = 0.0001). The burst pressure of the polymer with a Shore hardness of 85 exceeded even that of bovine pericardium (1808 ± 181 kPa vs. 1303 ± 176 kPa, *p* = 0.0002). With regards to the different polymers tested, polymers having a Shore hardness of 30–60 had similar burst pressures (ANOVA, *p* = 0.4281). Taken together, all polymers having a Shore hardness of 30–85 proved to resist a burst pressure comparable to aortic tissue.

## 4. Discussion

The use of 3D-printed vascular models is increasingly common, in order to address several key limitations inherent in conventional training methods, including constraints regarding the availability of cadaveric or animal tissues, ethical and hygienic concerns, and biomechanical inconsistencies [24,25,26,27,28]. With regards to animal tissues, substantial biomechanical disparities emerge when compared to human vessels, including nonlinearity, elasticity and stiffness, thereby constraining their efficacy in creating realistic surgical simulation and training models [17]. In contrast, 3D-printed models offer several advantages, including reproducibility, patient-specific customization, mechanical stability, and the elimination of hygienic concerns. These features render them highly advantageous for a variety of applications, such as training, device testing, and preclinical research [7,29,30,31,32,33,34,35].

A major advantage of PolyJet printing is the availability and versatility of PolyJet photopolymers, which enable precise adjustment of mechanical properties like elasticity, hardness, and tensile strength [36,37]. Agilus^®^- [36,38] and Tango^®^ [25,32,39]-based materials have been tested and shown to effectively reproduce nonlinear and, to a certain extent, anisotropic mechanical properties relevant to human aortic tissue simulation [31,36,40]. The capacity for multi-material printing serves to broaden the scope of these properties, thereby enabling the fabrication of layered composites that exhibit a high degree of similarity to intricate biological structures [41,42]. Kornfellner et al. [40] and Zhalmuratova et al. [41] demonstrated that such multi-material and fabric-reinforced elastomeric constructs can simulate the nonlinear, anisotropic stress–strain behavior of native human or porcine aorta. These properties, together with their proven high-resolution printing accuracy [43], support their use in the generation of high-fidelity vascular phantoms [40,41]. Taking into account the results of other groups evaluating applications of the Agilus^®^ and Tango^®^ polymers, their use in the generation of phantoms for simulation purposes has been positively evaluated [38,44,45]. Of note is that the phantoms generated in this study are intended neither to have contact with the human body nor to be implanted into the human body. However, no information regarding long-term durability, environmental stability and performance under humidity, temperature cycling, or UV exposure is disclosed in the manufacturer’s datasheet.

Notably, additional polymers like Alginate/PEG [46] or SLA-polymer elastic resin 50A/80A (Formlabs) [47] have been developed or became available that facilitate 3D printing of flexible aortic phantoms. Those materials were shown to exhibit mechanical properties comparable to those of aortic tissue. Furthermore, 3D-printable silicone has been positively evaluated and shown to have tensile strength and suture behavior comparable to aortic tissue [21].

The selection of porcine aorta and bovine pericardium as control materials was based on their availability, established use in cardiovascular surgery, and extensively characterized mechanical properties. Porcine aorta is commonly used as a surrogate for human aortic tissue due to its comparable geometry, fiber orientation, and structural behavior under load, particularly in younger specimens [17]. However, several studies have shown that aged human aortic tissue is significantly stiffer and exhibits more nonlinear behavior, limiting direct translatability [41,48]. Bovine pericardium is a routinely used graft for reconstructive procedures in vascular surgery and is characterized by its high tensile strength, durability, and biocompatibility [49,50]. Nevertheless, its stiffness and collagen density derived from a fixation process is higher than native aortic tissue and can lead to mismatches in mechanical compliance when used as a reference material [51]. Despite these limitations, both tissues remain widely accepted standards in benchmark testing.

To evaluate the performance of different polymers under physiological stresses encountered in vascular surgical applications, standardized approaches for biomechanical testing, including tensile strength, burst pressure resistance, and suture retention strength, were chosen. Tensile testing assesses resistance to stretching forces. Burst pressure testing determines failure pressure thresholds, and suture retention strength measures secure suture anchoring, all key features of surgical suitability [21]. Existing literature on the mechanical testing of PolyJet photopolymers primarily supports their potential as vascular tissue analogues by demonstrating successful replication of biomechanical properties, such as tensile strength and elasticity [11,36,37]. However, burst pressure testing of PolyJet-printed materials has not been reported in the current literature, and therefore, the inclusion of burst pressure testing represents a novel aspect of our study. Our results for burst pressure testing for bovine pericardium as a standardized test material compare well to the results obtained by others [52].

Assessment of suture retention strength of a single stitch demonstrated that all tested polymers (ShA 27–85) were non-inferior to porcine aortic and bovine pericardial tissue, highlighting their suitability for surgical handling and simulation. Notably, intact polymers within the ShA 30–50 range exhibited mechanical characteristics, such as maximum force, tensile strength, strain energy, and burst pressure resistance, that were comparable or even superior to native aortic tissue. These findings extend current literature, including the study by Illi et al., who reported that PolyJet photopolymers were generally stiffer than porcine tissue and lacked the nonlinear stress–strain response typical of biological vessels [17,50]. In contrast, our findings revealed that the polymers tested, while still more elastic (i.e., with lower elastic modulus), offered significantly greater elongation at break, which may enhance deformation behavior under load. Lin et al. similarly noted challenges in replicating the compliance and anisotropy of native tissue using current photopolymers, yet acknowledged improvements in novel formulations [53]. Our results support this by identifying specific polymers, particularly those with ShA 30–50, that remain mechanically resilient even after suturing. While suturing reduced tensile strength and strain energy, these polymers retained non-inferior performance compared to sutured biological tissues. In contrast, TangoPlus (ShA 27) showed inferior performance, confirming the need for a minimal Shore hardness threshold to ensure mechanical reliability for vascular phantom applications.

Several limitations of this study must be considered. First, since human aortic tissue is not readily available as a control material, the use of porcine aortic tissue and bovine pericardium as substitutes was necessary. Although these xenogenic tissues share biomechanical characteristics with human tissues, significant differences, such as higher stiffness and distinct nonlinear and anisotropic behaviors, potentially limit the direct comparison and translation of these results. Second, the mechanical tests conducted in this study were primarily uniaxial and static in nature, i.e., they do not fully replicate the dynamic and complex biaxial loading conditions encountered in vivo. Such simplifications may limit the comprehensiveness of mechanical characterization of the viscoelastic and anisotropic behavior of the polymers and therefore could potentially underestimate or overlook certain biomechanical aspects relevant in clinical scenarios. Last, this investigation was not designed to explore the long-term durability of the studied polymers, a factor that is critical to their potential application in surgical simulations. Subsequent research could therefore address biaxial and extended durability testing to confirm long-term mechanical stability and further define the suitability of these materials for modelling purposes.

## 5. Conclusions

This is the first report to provide a head-to-head comparison of Tango^®^ and Agilus30^®^ polymers with regards to stress–strain and burst pressure testing. Flexible polyjet polymers having a Shore hardness of 30–50 most closely mimic the mechanical properties of aortic tissue and can therefore be employed for additive manufacturing (3D printing) of aortic phantoms for simulation and training purposes. These findings support their suitability for use in medical simulation, surgical training, and procedural planning. Further advancements and material optimizations are expected to enhance the biomechanical realism and functional fidelity of vascular models, ultimately paving the way towards patient-specific precision medicine.

## Data Availability

Data is contained within the article. Further inquiries can be directed to the corresponding author.
